# Response to the Comments from the Groupe Francophone de Cytogénétique Hématologique (GFCH) on the 5th edition of the World Health Organization classification of haematolymphoid tumors

**DOI:** 10.1038/s41375-023-01872-6

**Published:** 2023-03-27

**Authors:** Reiner Siebert, Anna Schuh, German Ott, Ian A. Cree, Ming-Qing Du, Judith Ferry, Andreas Hochhaus, Kikkeri N. Naresh, Eric Solary, Joseph D. Khoury

**Affiliations:** 1 Institute of Human Genetics, Ulm University and Ulm University Medical Center, Ulm, Germany; 2 Department of Oncology, University of Oxford, Oxford, UK; 3 Department of Clinical Pathology, Robert-Bosch-Krankenhaus, and Dr. Margarete Fischer-Bosch Institute of Clinical Pharmacology, Stuttgart, Germany; 4 International Agency for Research on Cancer (IARC), World Health Organization, 25 Avenue Tony Garnier, CS 90627, 69366 Lyon, France; 5 Division of Cellular and Molecular Pathology, Department of Pathology, University of Cambridge, Cambridge, UK; 6 Department of Pathology, Massachusetts General Hospital and Harvard Medical School, Boston, MA USA; 7 Hematology/Oncology, Universitätsklinikum Jena, Jena, Germany; 8 Section of Pathology, Clinical Research Division, Fred Hutchinson Cancer Center, Seattle, WA USA; 9 Department of Hematology, Gustave Roussy Cancer Center, Université Paris-Saclay, Villejuif, France; 10 Department of Pathology and Microbiology, University of Nebraska Medical Center, Omaha, NE USA

**Keywords:** Haematological cancer, Lymphoma, Leukaemia

## To the Editor:

We thank Nguyen-Khac et al. [1]. who, on behalf of the Groupe Francophone de Cytogénétique Hématologique (GFCH), provide thoughtful comments on the summary of the 5th Edition of the World Health Organization (WHO) Classification of Haematolymphoid Tumors (WHO-HAEM5) [2, 3] and express that the new edition is a reflection of “*the most up-to-date-knowledge on the topic*”. The comments in the Letter to the Editor touch on several important aspects of WHO-HAEM5, with emphasis on genetic aspects of the classification. As a representative group of editors who primarily oversaw topics raised in the Letter, we provide this response on behalf of the rest of our esteemed colleagues who contributed to the classification. We take the opportunity to also add some explanatory comments on certain issues tangentially related to the topics raised by the GFCH.

## Chromosomal abnormalities in disease names

The GFCH states that “*chromosomal abnormalities […] have been removed from the WHO-HAEM5 classification of most of the hematological malignancies with defining genetic abnormalities caused by this type of defect*—*especially acute leukemia*”. We would like to clarify that although the nomenclature of genetically defined entities in WHO-HAEM5 no longer includes chromosomal translocations, the importance of chromosomal abnormalities and their detection using chromosomal banding analysis has not been diminished at all. Indeed, chromosomal abnormalities are discussed in detail in essentially all sections in which they are relevant, as can be seen in the beta version of the classification available online (https://tumourclassification.iarc.who.int). We completely agree with the GFCH that chromosome banding analysis continues to contribute fundamental knowledge in the management of patients with hematological cancers. Indeed, WHO-HAEM5 consistently emphasizes the notion that a variety of diagnostic tools may be helpful to detect a particular disease-defining abnormality. However, on balance, the WHO-HAEM5 editorial board determined that including chromosomal abnormalities alongside disease-defining genetic alterations in the name of a disease type would add unnecessary complexity and redundancy.

## Fusion and other terminology used for deregulated genes

The GFCH proposes that the term “juxtaposition” is more suitable than “fusion” when referring to the deregulation of an intact oncogene. Here we should note that this comment effectively takes issue with the guidelines of the HUGO Gene Nomenclature Committee (HGNC) [4], which is independent from WHO-HAEM5 but has been adopted as a standard therein. Notwithstanding, we do agree with the GFCH that the HGNC recommendations in this regard lead to the loss of important information, as different pathogenetic mechanisms cannot be distinguished. Namely, alterations leading to bona fide fusion genes, i.e., a fusion transcript translated into a fusion protein from an in-frame link of two partial genes, are not differentiated from enhancer-hijacking mechanisms that juxtapose a full coding region of a physiologically existing gene next to the regulatory regions of another gene resulting in dysregulated expression of the gene and its encoded full-length protein. This distinction is certainly highly meaningful from a cancer genetics and molecular pathogenesis point of view. Alas, such genetic precision is often lost in routine practice. For example, commercial assays for the detection of enhancer-hijacking mechanisms use the term “fusion assay” (like dual-color dual fusion FISH assays e.g., for the named *IG*::*MYC* and *IGH*::*CCND1* “fusions”). In addition, some enhancer-hijacking mechanisms, like several *IGH* translocations, have been shown to express chimeric transcripts joining both partner loci, though these are not bona fide gene fusions. Thus, the term “gene fusion” has been adopted in the WHO Classification as an umbrella term for structural chromosomal variants contributing to oncogenesis through bona fide gene fusion or enhancer-hijacking mechanisms.

Along similar lines, we acknowledge that the term “rearrangement” is applied more widely than defined *sensu strictu*. The term “rearrangement” narrowly defined refers to the genomic assembly of gene segments to a functional gene as typical for *IG* and *TR* loci. In this regard, reporting *IG* or *TR* rearrangements always needs to include information on the pattern of rearrangement, i.e., monoclonal, oligoclonal or polyclonal. Acknowledging the terminology commonly used in daily practice, here also the WHO-HAEM5 adopts the term “rearrangement” as an umbrella term for all kinds of pathogenic structural genomic variants affecting a given gene, including chromosomal translocations, inversions, copy number variants, etc. As this can lead to some ambiguity regarding the type of genomic variants (e.g., changes in gene dosage vs. gene regulation) and their consequences (e.g., bona fide gene-fusion vs. enhancer hijacking), the reader is referred to the exact definitions of the meaning for a given entity within respective sections of the Blue Book.

We would like to take the opportunity to also point that the WHO-HAEM5 follows the HGNC guidelines regarding italicizing immunoglobulin (*IGH, IGK, IGL*) and T-cell receptor (*TRA, TRB, TRD, TRG*) loci [4]. In contrast, mutated gene products, i.e., mutated proteins, are not italicized.

## Clonal cytopenia of undetermined significance (CCUS) and myelodysplastic neoplasms (MDS)

Formal inclusion of clonal haematopoiesis of indeterminate potential (CHIP) and clonal cytopenia of undetermined significance (CCUS) as distinct disease types in WHO-HAEM5 provides an opportunity to improve the demarcation between these precursor conditions and myelodysplastic neoplasms (MDS). Within this framework, CCUS includes cases with somatic mutations in myeloid malignancy-associated genes meeting the criteria of CHIP and/or clonal chromosomal abnormalities in a patient with one or more cytopenia(s) but without morphologic dysplasia. On the other hand, the prerequisites for the diagnosis of MDS fundamentally include cytopenia(s) and morphologic dysplasia. The authors and editors of WHO-HAEM5 contend that this framework will continue to identify patients with bona fide MDS, while avoiding over-diagnosis of patients with precursor myeloid states and rare cases of transient clonal chromosomal abnormalities [5].

The CCUS/MDS diagnostic framework in WHO-HAEM5 does align in principle with the proposal by Brett et al. [6]. Indeed, WHO-HAEM5 establishes that any non-recurrent clonal abnormality may define CCUS in the absence of morphologic dysplasia. WHO-HAEM4R and other more recent proposals have made exceptions to this premise and allow bypassing the dysplasia requirement when certain cytogenetic alterations are detected, including: complex karyotype; 5q deletion or loss of 5q due to unbalanced translocation; monosomy 7, 7q deletion, or loss of 7q due to unbalanced translocation; or inv(3q)/t(3;3). The WHO-HAEM5 editors and authors contend that such an exception is unnecessary, since cases harboring such abnormalities hardly ever occur without concomitant dysplasia and other clear stigmata of MDS. Indeed, the detection of such abnormalities in a patient who does not clearly meet the diagnostic criteria for MDS should raise concern and prompt careful diagnostic evaluation and close follow up. The detection of inv(3q)/t(3;3) should prompt assessment for *MECOM* rearrangement, which, if present, would support a diagnosis of acute myeloid leukemia with *MECOM* rearrangement. We do concur with the authors that it would not be practical to refer to clonal abnormalities in “myeloid cells”.

## B-cell prolymphocytic leukemia

The authors raise several questions regarding B-cell prolymphocytic leukemia (B-PLL) and raise concern that it is no longer recognized as an entity in WHO-HAEM5. Nguyen-Khac et al. [1] would not be surprised to know that this topic generated vivid and thoughtful discussions. In the end, the consensus among oncologists, haematopathologists, and geneticists was that B-PLL lacked sufficient distinction to warrant retaining it as a distinct disease type. We acknowledge of course that WHO-HAEM4R had already removed MCL with nucleolated cells resembling prolymphocytes from B-PLL. This left CD5-positive B-cell lymphoid proliferations with >15% prolymphocytes divided into two categories: atypical CLL (CLL/PLL) with ≤55% prolymphocytes; and B-PLL with >55% prolymphocytes. The latter category is also heterogeneous; it includes cases that have prolymphocytes that are morphologically identical to those seen in CLL and other cases characterized by a uniform population of medium to large lymphoid cells with prominent nucleoli. Here it should be noted that the 55% prolymphocyte count used to distinguish between atypical CLL and B-PLL had been set arbitrarily and its value is likely impossible to validate at present. Moreover, so-called B-PLL cases were highly heterogeneous in terms of immunophenotype and genetic features. *MYC* aberrations, *TP53* disruption, trisomy 12, del(8p), and under-representation of del(13q) show strong resemblance to poor-risk CLL, albeit they remain distinct from diffuse large B-cell lymphoma-type Richter transformation [7]. Together, these findings support classifying such cases as prolymphocytic transformation of CLL.

This leaves a group of CD5-negative, mostly splenic, lymphomas with prominent nucleoli that could not be grouped with more common diseases. These were grouped together as a new “placeholder” entity called *splenic B-cell lymphoma/leukemia with prominent nucleoli* (SBLPN). This new entity includes some cases of splenic marginal zone lymphoma, CD5-negative cases of B-PLL and HCLv. The rationale for including HCLv in SBLPN is that it classically shows prominent nucleoli but does not have clinical, immunophenotypic, or genetic features of HCL. We acknowledge that this is based on the evaluation of only a total of 15 cases with HCLv [8]. Of these, five had mutation and/or deletion of *TP53* compared to none in HCL. The presence of *TP53* mutation/deletion was strongly associated with poor outcomes in these patients, making TP53 the most clinically relevant disease driver in this entity. Interestingly, cases formerly labelled as CD5-negative B-PLL also have prominent nucleoli with frequent *TP53* disruption conferring poor prognosis. *TP53* and *MYC* alterations are classically associated with progression and/or transformation, and their frequent occurrence in SBLPN may point to a category of “progressed” lymphomas distinct from “transformation”, as discussed in the Blue Book section on transformed indolent lymphomas.

Our approach to B-PLL and SBLPL has aimed to be systematic and evidence-based while also being pragmatic. Namely, we had to consider the clinical perspective that splitting rare poor-risk patient groups into distinct disease entities adds barriers to clinical trial design and eligibility, whereas relating them—where appropriate—to more established entities (such as MCL, CLL) may ultimately fill the evidence gap. These classification challenges reflect the inherent difficulty in deriving clear disease types from small patient groups and heterogeneous genetic studies. In this context, we acknowledge that SBLPN is best regarded as a placeholder type that will require additional studies to identify distinctive features that could inform future editions of the classification.

## Concluding Remarks

In conclusion, we thank the GFCH for highlighting the value of WHO-HAEM5 classification and for raising important issues worthy of continued discussions. Our response is intended to provide some clarification regarding the points raised and shed light on some related decision processes that shaped the WHO-HAEM5 classification. We would like to conclude by pointing the GFCH and the readers to additional details in the “epilogue” of the summary paper by Alaggio et al. [3] in which we discuss the limitations and the opportunities of any classification, including WHO-HAEM5.

## Disclaimer

The content of this paper represents the personal views of the authors and does not represent the views of the authors’ employers and associated institutions. This work is intended to provide a preview and summary of content whose copyright belongs solely to the International Agency for Research on Cancer/World Health Organization. Any or all portions of the material in this work may appear in future International Agency for Research on Cancer/World Health Organization publications.

## References

[1] Nguyen-Khac F, Bidet A, Troadec MB, Veronese L, Auger N, Daudignon A, et al. The 5th edition of the WHO classification of haematolymphoid tumors: comments from the Groupe Francophone de Cytogénétique Hématologique (GFCH). Leukemia (in press) 2023. 10.1038/s41375-023-01821-3 36707618

[2] Khoury JD, Solary E, Abla O, Akkari Y, Alaggio R, Apperley JF (2022). The 5th edition of the World Health Organization Classification of Haematolymphoid Tumours: Myeloid and Histiocytic/Dendritic Neoplasms. Leukemia.

[3] Alaggio R, Amador C, Anagnostopoulos I, Attygalle AD, Araujo IBO, Berti E (2022). The 5th edition of the World Health Organization Classification of Haematolymphoid Tumours: Lymphoid Neoplasms. Leukemia.

[4] Bruford EA, Antonescu CR, Carroll AJ, Chinnaiyan A, Cree IA, Cross NCP (2021). HUGO Gene Nomenclature Committee (HGNC) recommendations for the designation of gene fusions. Leukemia.

[5] Gur HD, Wang SA, Tang Z, Hu S, Li S, Medeiros LJ (2017). Clinical significance of isolated del(7p) in myeloid neoplasms. Leuk Res.

[6] Brett VE, Lechevalier N, Trimoreau F, Dussiau C, Dimicoli-Salazar S, Coster L (2023). The presence of a chromosomal abnormality in cytopenia without dysplasia identifies a category of high-risk clonal cytopenia of unknown significance. Genes Chromosom Cancer.

[7] Chapiro E, Pramil E, Diop M, Roos-Weil D, Dillard C, Gabillaud C (2019). Genetic characterization of B-cell prolymphocytic leukemia: a prognostic model involving MYC and TP53. Blood.

[8] Hockley SL, Morgan GJ, Leone PE, Walker BA, Morilla A, Else M (2011). High-resolution genomic profiling in hairy cell leukemia-variant compared with typical hairy cell leukemia. Leukemia.

